# The Impact of Air Pollution on Cardiovascular Health Outcomes in African Populations: A Scoping Review

**DOI:** 10.1016/j.jacadv.2024.101371

**Published:** 2024-11-13

**Authors:** Marvellous Adeoye, Shadi Rahimzadeh, Sean Taylor, Shreya Shrikhande, Pablo Perel, Anoop Shah, Mariachiara Di Cesare, Mark R. Miller

**Affiliations:** a Institute of Public Health and Wellbeing, University of Essex, Colchester, United Kingdom; b Science and Public Health Department, World Heart Federation, Geneva, Switzerland; c Department of Non-Communicable Disease Epidemiology, London School of Hygiene & Tropical Medicine, London, United Kingdom; d Centre for Cardiovascular Science, University of Edinburgh, Edinburgh, United Kingdom

**Keywords:** Africa, air pollution, cardiovascular disease, public health

## Abstract

**Background:**

Air pollution is a significant environmental risk factor for cardiovascular diseases (CVDs), but its impact on African populations is under-researched due to limited air quality data and health studies.

**Objectives:**

The purpose of this study was to synthesize available research on the effects of air pollution on CVDs outcomes in African populations, identify knowledge gaps, and suggest areas for research and policy intervention.

**Methods:**

A systematic search of PubMed was conducted using terms capturing criteria ambient air pollutants (for example particulate matter, nitrogen dioxide, ozone, and sulfur dioxide) and CVDs and countries in Africa. Exclusions were studies on tobacco smoking, household air pollution, and occupational exposures.

**Results:**

Six studies met the full inclusion criteria. Most studies were conducted in urban settings and most investigated on particulate matter, nitrogen dioxide and sulfur dioxide. Five of the 6 studies were performed in South Africa. The studies showed positive associations between exposure to air pollutants and increased incidence of stroke and overall cardiovascular hospitalization and mortality. However, there was considerable variation in study design, pollutant measurement methods, and adjustment for confounders.

**Conclusions:**

This review highlights a critical need for standardized research on air pollution and cardiovascular health in Africa. The extremely limited numbers of studies make it difficult to ascertain the true impact of air pollution across the African continent. Future research should include longitudinal studies in different African populations with standardized methods. There is an urgent need to improve pollution monitoring networks, ascertain key sources of exposure, and implement air quality standards.

Air pollution stands as the world's foremost environmental risk factor for human health.^1^ Furthermore, the significant cardiovascular effects of air pollution are now well established, and there is growing recognition that air pollution has adverse effects on all organ systems of the body.^2^ Complex biological pathways involving inflammation, oxidative stress, and dysfunction of the vascular endothelium and thrombosis are key mechanisms underlying these adverse health impacts.^3^, ^4^, ^5^ Major air pollutants include particulate matter (PM), particularly PM_2.5_ (particles with an aerodynamic diameter <2.5 μm), nitrogen dioxide (NO_2_), sulfur dioxide (SO_2_), and ozone (O_3_).^6^

Africa, with a population exceeding 1 billon faces a concerning rise in cardiovascular diseases (CVDs), with projections suggesting they will surpass infectious diseases as the leading cause of death by 2030.^7^ Additionally, data from the World Health Organization estimate that ambient air pollution contributed to 4.2 million premature deaths worldwide in 2019.^8^ In the same year in Africa, air pollution was estimated to cause 780,000 deaths and 20.3 million disability-adjusted life years, with CVDs contributing to approximately halve of those number (334,000 deaths and 11.4 million disability-adjusted life years).^1^ While the continent shares the global challenge of air pollution, the drivers of cardiovascular morbidity and mortality are considerably different compared to high-income countries. Most African countries are characterized by a predominant burden of cerebrovascular disease, cardiomyopathies, and rheumatic heart disease and emerging HIV-related CVD; while in the more affluent areas of the region, the burden is characterized by hypertensive heart disease and related heart failure and emerging incidence of coronary artery disease, peripheral artery disease, and atrial fibrillation.^9^ This escalating burden coincides with limited access to quality health care and a high prevalence of co-existing chronic and infectious diseases in the region.^10^

The sources of air pollutants in Africa are varied, with significant contributions from the widespread use of biomass for cooking and heating and open burning of agricultural waste.^11^ These practices release high levels of PM_2.5_ and other harmful pollutants, exacerbating the cardiovascular health burden in the region. Despite the well-documented health effects of air pollution globally, significant research gaps exist in Africa. The continent faces unique challenges such as rapid urbanization and increasing motorization with estimates of transport-related emissions accounting for 40% of air pollution sources. Additionally, the interaction of natural dust storms with anthropogenic pollution adds complexity to understanding exposure-response relationships.^12^ The lack of region-specific data hampers the development of targeted public health interventions.

Air quality monitoring data in Africa, while scarce, reveal elevated air pollution levels in urban areas, creating uncertainty about the health effects compared to studies in other regions of the world. IQAir's 2023 World Air Quality Report provides a comprehensive overview and valuable insights into global air quality trends.^13^ The report sheds light on the significant gap in air quality monitoring systems across the African continent. Out of 54 countries, only 24 have the capacity to monitor air quality in some capacity, resulting in a limited data set. Also, the report highlights that only 2 South African cities, Nieuwoudtville and Bot River, in Africa met the World Health Organization PM_2.5_ air quality guidelines. Figure 1 highlights the deficiency of air monitoring stations in Africa, with most of the existing stations concentrated in the western and southern regions.^13^ Even where monitors exist, they do not measure all air pollutants that are considered key to health. In regions where ground-based monitoring is inadequate or absent, satellite data offer a promising alternative for estimating air pollution levels. Satellites can provide extensive coverage and continuous data on key pollutants like PM_2.5_ and NO_2_. While satellite data can bridge some of the gaps in monitoring, it also has limitations, such as low spatial resolution and assumptions that ground-level pollutants will reflect the column of air.^14^ However, with advancements in satellite technology and data processing, it holds potential for improving air quality assessments in Africa, especially in remote and underserved areas.

**Figure 1:**
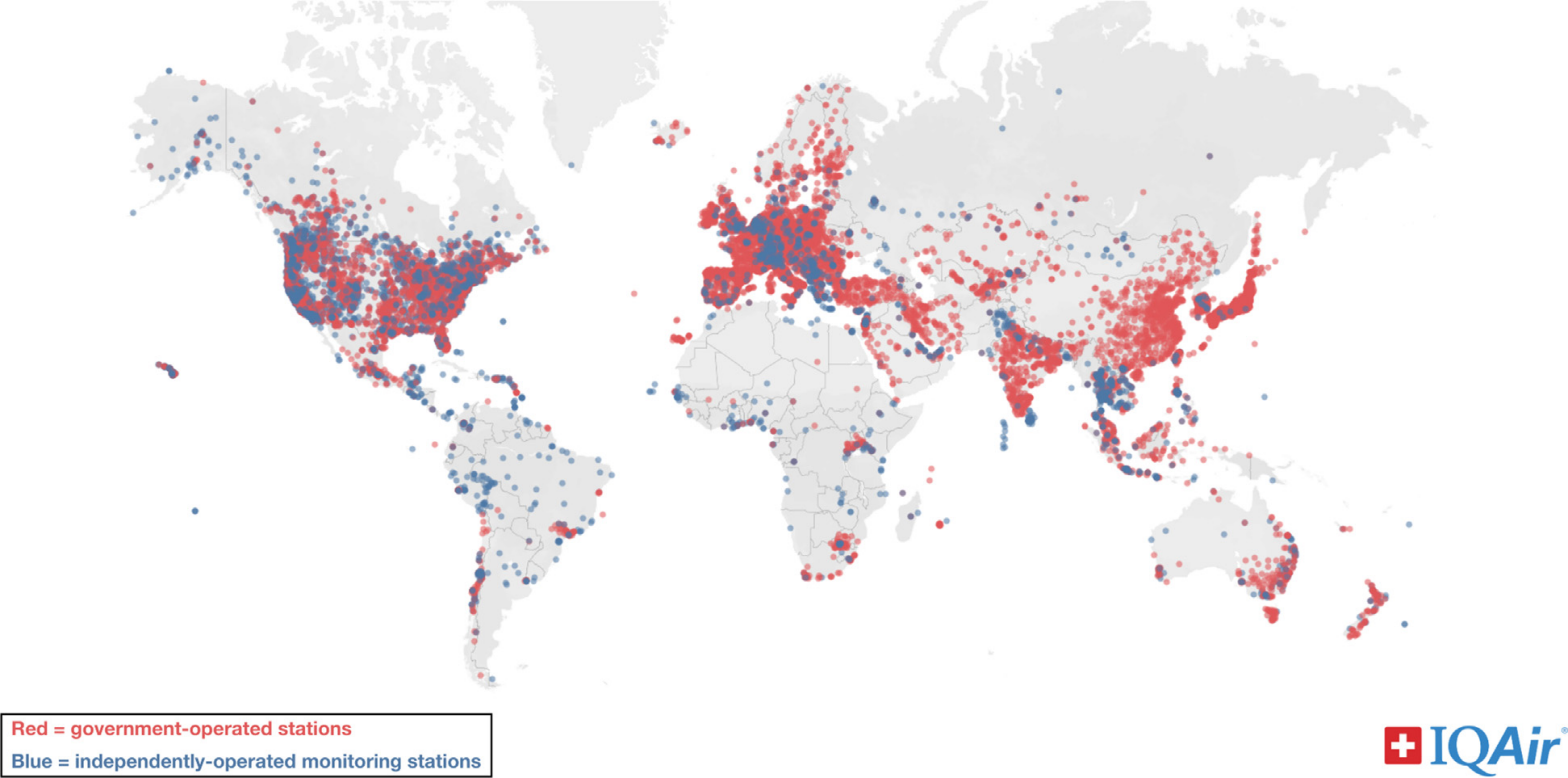
**Global Distribution of “Particulate Matter With a Diameter of 2.5 μm or Less” Monitoring Stations** Global distribution of PM_2.5_ monitors across the world. The map displays a significant disparity in the number of monitoring stations, with Africa having fewer monitors compared to other regions of the world. PM_2.5_ = particulate matter with a diameter of 2.5 μm or less.

Within this context, understanding the association between air pollution and CVDs in Africa becomes crucial. While evidence demonstrates a clear correlation between exposure to ambient air pollution and an increased risk of cardiovascular and metabolic conditions at a global level,^15^^,^^16^ data from African populations is scarce. A recent systematic review by Toubasi et al^17^ analyzing data from over 18 million ischemic stroke cases exemplifies this gap, revealing associations between NO_2_ and particulate matter with a diameter of 10 μm or less (PM_10_) with stroke mortality, but lacking any studies conducted in Africa. There is a need to investigate the applicability of exposure-response functions derived from high-income countries to low-income settings prevalent across Africa, especially for pollutants such as PM_2.5_ which can vary significantly in terms of source, size distribution, and chemical composition in different regions.

Given the escalating burden of CVDs in Africa, potential underestimation of air pollution's health effects, and limited existing research from the continent, this scoping review aims to comprehensively evaluate the current literature. We systematically map the existing body of research regarding the association between air pollution and cardiovascular health in Africa (Central Illustration). This review will identify key concepts, types of evidence available, and highlight critical gaps in knowledge to inform future research directions and guide targeted interventions to mitigate this growing public health crisis.

**Central Illustration:**
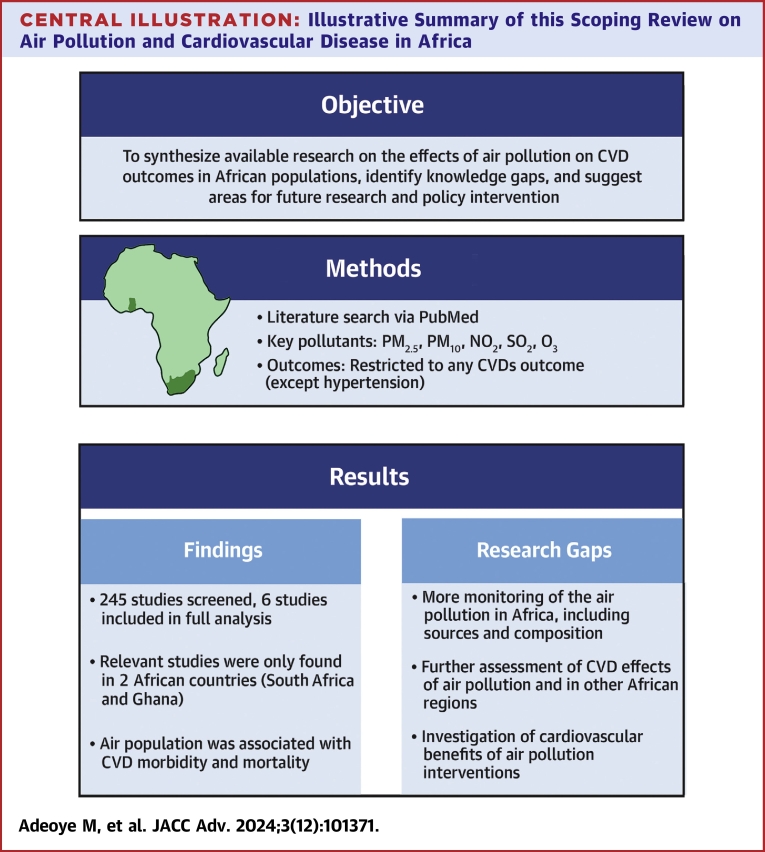
Illustrative Summary of this Scoping Review on Air Pollution and Cardiovascular Disease in Africa

## Methods

The methodological procedures followed in this study adhere to the established framework for scoping reviews.^18^^,^^19^ We followed the Preferred Reporting Items for Systematic Reviews and Meta-Analyses extension for Scoping Reviews (PRISMA-ScR).^20^

### Search strategy and database sources

The literature database PubMed (MEDLINE) was searched with the aim of identifying original research articles written in English from inception to October 21, 2023. The terms “air pollution,” “particulate matter,” “nitrogen dioxide,” “sulfur dioxide,” “ozone,” and their conjunctions with “cardiovascular diseases,” “cardiovascular mortality,” and “cardiovascular hospitalization,” alongside region-specific MESH terms like “Africa” and “African countries” were used (see Supplemental Appendix for complete search strategy).

### Eligibility

Included studies were those investigating pollutant levels within any African country, provided the data were quantifiable. The criteria for selecting studies on health outcomes included populations of children, adults, or the elderly residing in Africa. The focus was on ambient air pollutants, particularly those categorized as “critical air pollutants” by the U.S. Environmental Protection Agency (carbon monoxide, NO_2_, SO_2_, O_3_, PM_10_, and PM_2.5_).^21^ These pollutants are the criteria ambient air pollutants in terms of human health, including cardiovascular health, in different regions of the world.^22^ The study design criteria included epidemiological studies of any design, such as cross-sectional, case-control, case-crossover, and cohort studies, while outcomes were restricted to any CVDs outcome (except those focusing on hypertension). CVDs of interest included various forms of heart disease, cerebrovascular diseases, and diseases of arteries, which were defined by International Classification of Diseases (ICD)-codes (see Supplemental Appendix). Exclusions comprised studies in non-African populations, studies solely assessing tobacco smoking-related pollution, household air pollution, or occupational exposures. Furthermore, studies not written in English, systematic reviews, editorials, and those primarily focusing on Saharan dust were excluded from the review as they fall outside the intended scope. Studies focusing on hypertension were excluded because this review aimed to investigate the link between ambient air pollutants and CVDs defined by ICD codes, whereas cardiovascular risk factors were deemed beyond the scope of the current work, and merit focused review.

### Validity assessment

The validity assessment tool adapted for this study has been described elsewhere.^23^ It builds upon the methods established in 2 prior tools: the Newcastle-Ottawa Scale and the Cochrane Collaboration's risk of bias tool.^24^

This assessment comprised 3 components: validation of CVDs occurrence (scored from 0 to 1), quality of air pollutant measurements (scored from 0 to 1), and extent of adjustment for confounders (scored from 0 to 3). Regarding validation of CVDs occurrence, we deemed the diagnosis validated if it was coded according to the ICD or based on clinical, laboratory, and imaging findings (a score of 0 was assigned in the absence of valid criteria). For assessing the quality of air pollutant measurements, we considered factors such as measurement frequency and the presence of missing data. A score of 0 was given if measurements were not conducted at least daily or if there was more than 25% missing data. Conversely, a score of 1 was assigned if measurements were conducted at least daily without exceeding 25% of missing data.

In evaluating adjustment for confounders, a score of 0 was allocated if no adjustments were made for long-term trends, seasonality, and temperature. A score of 1 was given if only these 3 adjustments were made. If additional adjustments were implemented, such as for humidity or day of the week, a score of 2 was assigned. A score of 3 was awarded if adjustments were made for influenza epidemics and holidays, in addition to those corresponding to a score of 2. Furthermore, if a study achieved the maximum score for all 3 components, it was categorized as being of good quality. Conversely, if one component scored the minimum (zero), the study was automatically classified as low quality. Studies that did not fall into either extreme category were considered of intermediate quality. For the cross-sectional study, the Quality Assessment Tool for Observational Cohorts and Cross-sectional Studies developed by the National Heart, Lung, and Blood Institute^25^ was used to supplement the quality assessment for the other studies (see Supplemental Appendix).

### Screening and data extraction

After conducting a comprehensive search, all identified results were entered into Endnote and duplicate entries were removed. For the screening of papers, the Rayyan web-based software was used. The review was done in 2 stages, starting with an initial evaluation based on titles and abstracts, followed by full-text screening. During the primary screening, M.A. and S.R. independently assessed titles and abstracts. Any disparities between the reviewers were deliberated, and if necessary, resolved by a third reviewer.

Two reviewers extracted essential characteristics from each included article using a predefined data-extraction sheet. The summary table included the following details: 1) study characteristics (including first author, publication year, study design, country/study location, population, and sample size); 2) characteristics related to ambient air pollution measurement (such as data type, type of pollutants, and measurement details); and 3) health outcomes (CVD morbidity and mortality).

Descriptive statistics were used to summarize the data by study location, measurement type, study design, and health outcomes.

### Ethical approval

As this study is a scoping review of existing literature, it did not involve any direct interaction with human or animal subjects and therefore did not require ethical approval.

## Results

**Figure 2:**
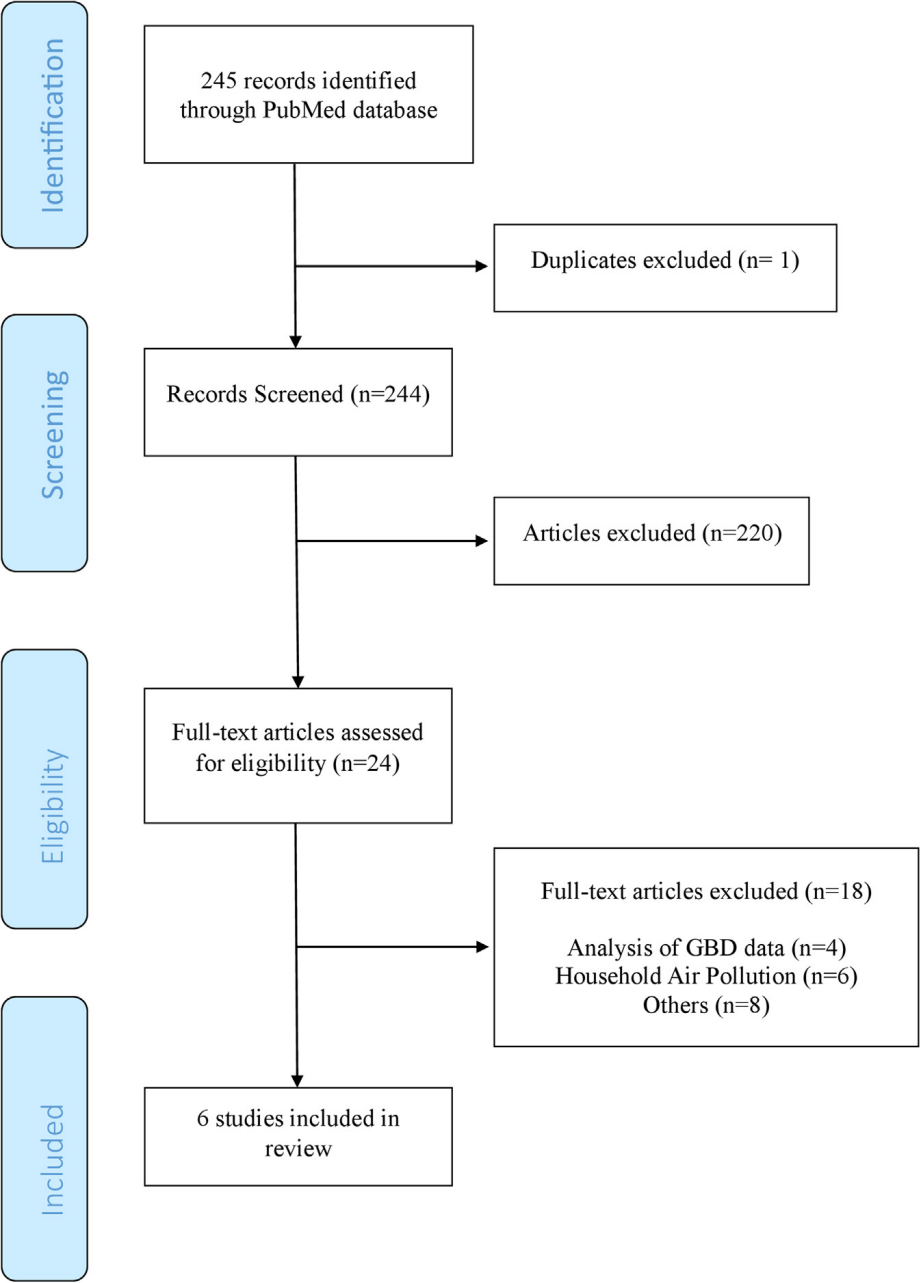
**PRISMA Flow-Chart of Study Selection** Systematic approach to study retrieval for the scoping review. GBD = Global Burden of Disease; PRISMA = Preferred Reporting Items for Systematic Reviews and Meta-Analyses.

A total of 244 papers were identified after deduplication, which were independently screened by 2 reviewers. Out of these, 24 papers were deemed suitable for a comprehensive examination of the full text. Major reasons for exclusion included the use of the Global Burden of Disease study that did not fit the eligibility criteria or were assessments of household air pollutants rather than ambient air pollutants. Ultimately, 6 papers^26^, ^27^, ^28^, ^29^, ^30^, ^31^ were identified and included in the review (Figure 2).

### General characteristics of the included studies

**Figure 3:**
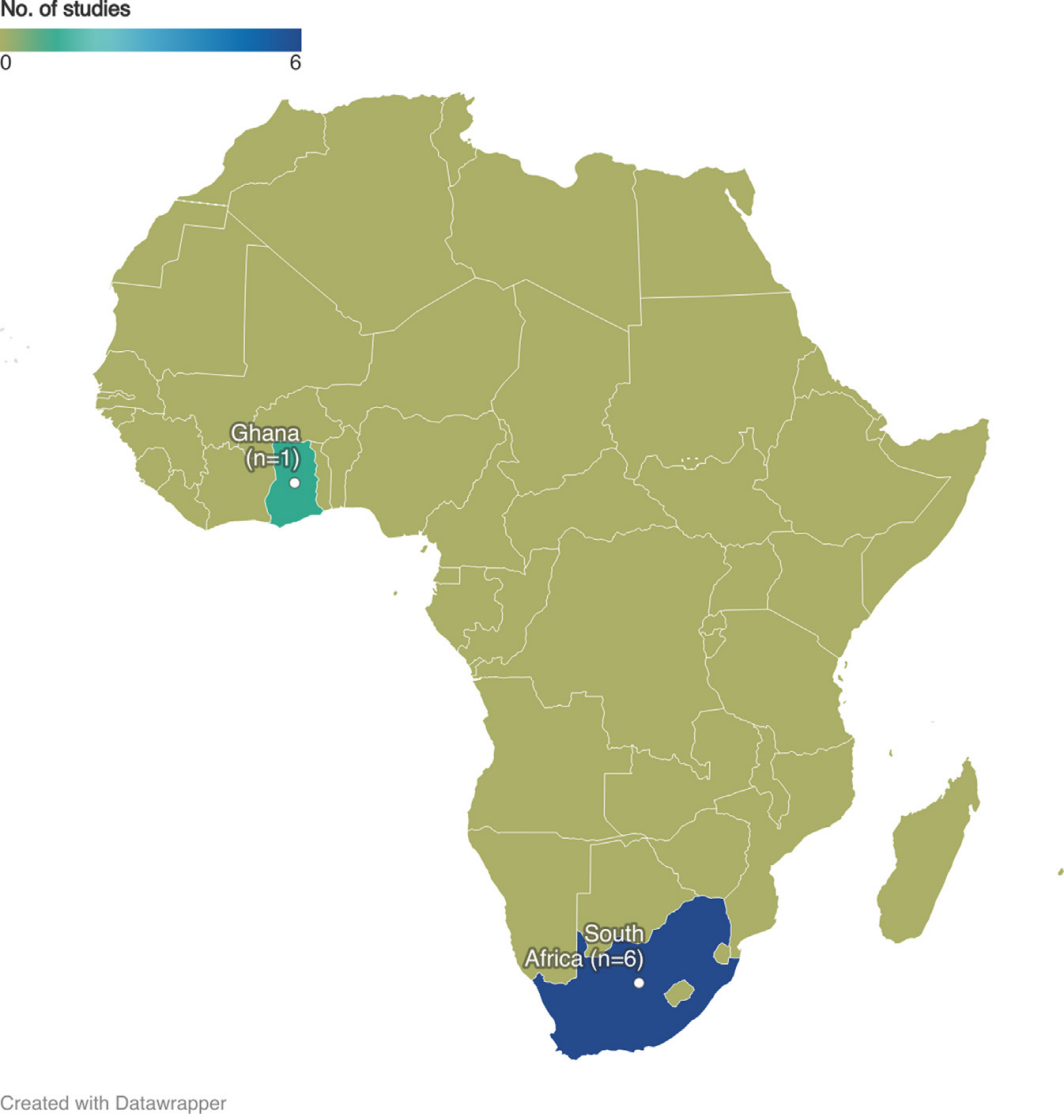
**Map Illustrating the Study Locations** Geographical distribution of studies across Africa. Out of the 6 studies, only one involved participant from Ghana, while the remaining 5 were primarily conducted in South Africa.

Table 1 outlines the characteristics of the 6 selected studies. Approximately two-thirds were published within the last decade, with more than half emerging in the past 5 years. Out of the 6 studies, 5 were conducted solely in South Africa, with Cape Town having the most number of studies (n = 4). The remaining study encompassed 6 low- and middle-income countries, including South Africa and Ghana (Figure 3). Among the 6 studies included in this review, 4^26^, ^27^, ^28^, ^29^ contained authors from the same research group. It is important to clarify that these studies were not derived from the same cohort; each study was conducted independently over different years and utilized data from various centers. However, references^27^ and^29^ both involve cohorts from 7 private hospitals in Cape Town, although the specific hospital names were not disclosed. The number of events recorded in both papers is identical, and some overall descriptive statistics are consistent between them, suggesting that the 2 papers likely used the same sample of cases. Despite this, the studies addressed different research questions and employed distinct study designs—time-series and case-crossover, respectively. In Table 1, we have combined these papers into a single row with a line demarcating their differences.

**Table 1:** General Characteristics of the Included Studies

First Author, Year	Year of Study	Town	Data Source	Study Design	Events (Total)	Age Included (y)	Pollutant	Concentration Increase μg/m^3^	Exposure Assessment	Outcome Ascertainment	Outcome
Wichmann et al, 2012^26^	2001-2006	Cape Town	Health Information and Technology department, City of Cape Town	Case-crossover	29,163	All age group	PM_10_, NO_2_, and SO_2_	12 μg/m^3^	Air quality monitoring laboratory	Health records coded according to ICD-10th Revision codes: (I00-I02), (I05-I09), (I10-I15), (I20-I25), (I26-I28), (I30-I43. I50, I51-I52), (I60-I69)	A significant increase of 3% in CVD mortality
Lin et al, 2017^31^	2007-2010	China, Ghana, India, Mexico, Russia, and South Africa	WHO Study on Global Ageing and Adult Health (SAGE), Wave 1	Cross-sectional/household surveys	45,625	≥18	PM2.5	10 μg/m^3^	Satellite data	Self-reported stroke history or diagnosed as stroke by a health care professional	Stroke (OR: 1.13; 95% CI: 1.04-1.22)
Adebayo-Ojo et al, 2022^28^	2006-2015	Cape Town	Statistics South Africa (StatsSA)	Time-series	54,356	≥15	PM_10_, NO_2_, SO_2_, O_3_	16.4 μg/m^3^ for PM_10_, 10.7 μg/m^3^ for NO_2_, 6 μg/m^3^ for SO_2_, and 15.6 μg/m^3^ for O_3_	Air quality monitoring network of 14 stations	Mortality data coded according to ICD-10 (I00-I99)	Increased CVD risks of 2.4% PM_10_, 2.2% NO_2_, 1.4% SO_2_ 2.5% O_3_
Roomaney et al, 2022^30^	2000, 2006, 2012	Multiple provinces	NA	Comparative risk assessment	NA	All age group	PM_2.5_, O_3_	NA	Ground monitoring stations	ICD-10th Revision codes; (I20-I25), (I60-I69)	CVD mortality attributable to PM_2.5_ 36% (2000) and 38% (2012)
Adebayo-Ojo et al, 2022^27^	2011-2016	Cape Town	7 private hospital	Time-series	54,818	All age group	PM_10_, NO_2_, SO_2_	12 μg/m3 for PM_10_, 7.3 μg/m^3^ for NO_2_, and 3.6 μg/m^3^ for SO_2_	Ground monitoring stations	Hospital records coded according to ICD-10th Revision (I00-I99)	Overall RR of CVD hospitalization risk 2.1% PM_10_, 1% NO_2_ and -0.3% SO_2_
Lokotola et al, 2020^29^				Case-crossover		≥15		10 μg/m^3^			Percentage change CVD hospitalization during warm periods for PM_10_ 6.3 (3.4, 9.3), NO_2_ 12.5 (5.3, 20.2), and SO_2_ 14.7 (5.0, 25.3)

CVD = cardiovascular disease; ICD = International Classification of Diseases; NA = not available; NO = nitric oxide; O_3_ = ozone; PM_2.5_ = particulate matter with a diameter of 2.5 μm or less; PM_10_ = particulate matter with a diameter of 10 μm or less; RR = relative risk; SO_2_ = sulfur dioxide; WHO = World Health Organization.

Of note, all study populations were centered in urban areas. The studies employed various design and analysis approaches: 2 used time-series designs with Poisson models, 2 employed case-crossover designs with conditional logistic regression, 1 used a cross-sectional design with a multilevel logistic regression model, and the final study conducted a comparative risk assessment.

### Types of pollutants

Five out of the 6 studies considered exposure from a combination of pollutants. The most used combination was PM_10_, NO_2_, and SO_2_ (4 studies). One of these also considered O_3_ along with PM_10_, NO_2_, and SO_2_. All 4 of these studies obtained air pollution data from government air quality monitoring registries. Two out of the 6 reviewed papers evaluated the impact of PM_2.5_ on cardiovascular health outcomes. One study focused solely on PM_2.5_, while another assessed it alongside O_3_.

#### Particulate matter with a diameter of 10 μm or less

Four studies that investigated the association between PM_10_ and CVD hospitalization and mortality outcomes found a positive association. Three studies quantified the risk of CVD outcomes for every IQR of PM_10_. For example, an IQR increase of 12 μg/m^3^ was associated with a 4% increase in the risk of cerebrovascular disease mortality^26^ and a 1.9% increase in the risk of CVD hospital admissions.^27^

Subgroup analyses in these studies also showed the effect of distance from the monitoring stations, temperature, sex, and age. For instance, those living within 10 km from the monitoring sites were more vulnerable to CVD mortality 4.3% (1.6% to 7.2%). Additionally, there was a stronger association between PM_10_ and CVD mortality in individuals aged over 65 years and females, with modeled risk increments of 3.3% and 3.2%, respectively.^28^ On the other hand, stronger effect estimates were seen with CVD hospital admission and PM_10_ among the 0 to 14 age group and males.^27^ All 4 studies controlled for the temperature or seasonal effects in their models. Three studies found a stronger association between PM_10_ and CVDs outcomes on warmer days, compared to normal or cold days.^26^^,^^27^^,^^29^

#### Particulate matter with a diameter of 2.5 μm or less

Only 2 studies examined the effect of PM_2.5_ on CVD outcomes, using different methods (cross-sectional and comparative risk assessment). Overall, both found PM_2.5_ exposure was positively associated with CVD outcomes. In the first study, researchers examined CVD deaths over a 12-year period and observed a 19.9% rise in the proportion of CVD deaths attributed to PM_2.5_ exposure.^30^ The second study found that the odds of stroke increased by a factor of 1.13 (with a 95% CI of 1.04-1.22) for every 10 μg/m^3^ increase in PM_2.5_ concentration.^31^

#### Nitrogen dioxide

Four out of the 6 studies examined NO_2_ in their analyses, with 1 study focusing on temperature as an effect modifier for the effect of NO_2_ on CVDs.^29^ Two studies found a positive association between NO_2_ and CVD mortality with similar estimates of a 3% increase in CVD mortality per IQR increase of 12 μg/m^3^ and 2.2% increase per IQR of 10 μg/m^3^.^26^^,^^28^ Temperature and other pollutants such as PM_10_ were found to act as effect modifiers for NO_2_ on CVD, with Adebajo-Ojo et al^28^ reporting a substantially reduced association when adjusted for PM_10_ and Lokotola et al^29^ and Wichmann et al^26^ reporting a stronger association during the warm periods.^26^^,^^28^^,^^29^ One of the studies which focused on CVD hospitalizations did not observe an association with NO_2_.^27^ The age and sex stratified analyses suggested that men below the age of 60 years were more sensitive to the increase in NO_2_ compared to females and people in the older age group.^26^^,^^28^

#### Sulfur dioxide

Temperature appeared to play a key role in modifying the association between SO_2_ and CVD outcomes, with a higher risk on colder days. Two studies estimated an increase in CVDs mortality by 3% per 8 μg/m^3^ increase in the IQR of SO_2_^26^ and 1.14% per 6 μg/m^3^ increase in the IQR, although this was not a significant association.^28^

#### Ozone

Only 2 studies considered O_3_ exposure, out of which only one examined its association with CVD outcomes. Adebajo-Ojo et al^28^ estimated an increase in the risk of CVD mortality by 2.5% (95% CI: 0.2%-4.8%) per IQR increase (15.6 μg/m^3^) of O_3_,^28^ with no significant differences across age or sex. They also found that O_3_ associations were not modified by other pollutants.

## Discussion

This scoping review was undertaken to describe the existing literature and identify any gaps on air pollution and CVDs in Africa. Our review found only 6 studies on this topic in Africa with all of them conducted in South Africa and just one with Ghana included along with South Africa. Despite growing concerns about the impact of air pollution on public health globally, there remains a dearth of comprehensive studies focusing on African populations.

African populations possibly suffer from a different CVD spectrum compared to other regions in the world.^32^ The definition of CVD was relatively heterogenous in the studies included in our systematic review. Five of the 6 studies used ICD-10 coding to define outcomes. Of these, 4 studies used a wider code list that encompassed right heart pathology and rheumatic heart disease. One study restricted the definition to ischemic heart disease and stroke, the rates of which may differ in the region compared to other settings.^32^ Overall, the use of the wider code list across the 4 studies does ensure that these studies have captured conditions that may be more prevalent in the African region.

The pollutants studied PM_2.5_, PM_10_, NO_2_, SO_2_, and O_3_ had adverse effects on cardiovascular health, consistent with global studies.^33^, ^34^, ^35^, ^36^ For example, this aligns with findings from an umbrella review of systematic reviews which suggests that ambient air pollution, particularly short-term exposures to PM_2.5_, PM_10_, and NO, is associated with increased risks of CVDs.^34^

The most commonly assessed pollutants found in our review were PM_10_, NO_2,_ and SO_2_, with only 2 studies considering PM_2.5_ and O_3_, the former relying on satellite measurements. While satellite data can offer valuable estimates of risk, it often lacks the resolution required for detailed analysis and makes assumptions regarding ground levels of pollutants.^37^ However, ground level urban air quality data in the region remain sparse, with the establishment of continuous monitoring networks impeded by challenges such as high costs and the technical expertise required for equipment maintenance.^38^ Monitoring data can be complemented by modeling approaches, although the accuracy of such models relies on a critical mass of air pollution monitors and robust emissions data, which may not be available in most African countries. Additionally, studies employing ground measurements did not include PM_2.5_ as it was only systematically measured in South Africa from 2012 onward, and much of the data collected in the years after 2012 contained missing data.^39^

Consequently, the dearth of available data on PM_2.5_ levels in Africa is reflected by the very few studies found in our review. This is a major omission, given that associations between air pollution and health tend to be greater and more consistent for PM_2.5_ compared to other pollutants in other regions of the world, and especially so for CVDs.^40^^,^^41^ Such associations have been observed not only in high-income countries but also in low- and middle-income countries facing some of the same socioeconomic challenges to those in Africa.^42^, ^43^, ^44^ However, there are clear regional disparities between different low- and middle-income countries^45^ therefore, studies are needed to provide African-specific context.

Studies in this review reported a modifying effect of temperature on the association between air pollution and CVD outcomes, suggesting that warmer temperatures amplify the detrimental impact of pollutants. This finding aligns with existing literature, which has observed more consistent associations between air pollution and cardiovascular risk during periods of high temperature.^46^^,^^47^ Notably, Lokotola et al^29^ and Adebajo-Ojo et al^28^ documented stronger associations between PM_10_ and NO_2_ exposure with CVD hospital admissions and mortality during warmer days. Similarly, Wichmann et al^26^ observed a more pronounced link between NO_2_ and stroke mortality in warmer periods. Both air pollution and extremes of heat change cardiovascular function through several shared biological mechanisms^48^ highlighting the potential synergistic effect of heat and air pollution on cardiovascular health. These observations raise broader discussions about the health impacts of climate change. While climate change and air pollution are distinct issues, they are closely intertwined, with climate change, for example, having the potential to contribute to air pollution through increases in airborne dust from dry land and the occurrence of wildfires, and the black carbon constituents of PM retaining heat in the lower atmosphere.^48^ There is concern in regions where these environmental stressors may be particularly prominent, such as Africa, or their consequences especially in vulnerable populations. Prevention should be a priority for both these issues; however, mitigation efforts are needed in the present. Pre-emptive monitoring of heat or pollution episodes can be employed, to allow health care systems to anticipate admissions and individuals to take steps to reduce heat and pollutant exposure, where possible. Awareness and infrastructural support are required for these interventions to be effective.

Two studies found in our review suggested an association between O_3_ and CVDs. Associations between O_3_ and cardiovascular morbidity and mortality have been observed in other regions of the world,^49^ albeit with a moderate degree of inconsistency.^50^ The heightened risk may be attributed to O_3_'s highly seasonal nature, with higher concentrations in summer due to photochemical formation of this pollutant. Additionally, O_3_ can undergo atmospheric reactions to alter the levels of other pollutants such as NO_2_, and potentially the oxidative reactivity of PM. Therefore, where possible, studies assessing the health effects of O_3_ should account for seasonal variations and address associations with 2 pollutant models.^51^^,^^52^

### Geographic disparities and urban focus

The included papers highlight a concentration of research in South Africa, particularly in Cape Town, revealing a significant dearth of studies in other countries. This finding is consistent with a narrative review on the health impacts of ambient air pollution in sub-Saharan Africa, which recognizes the abundance of monitoring stations in South Africa.^53^^,^^54^ It is important to note that South Africa's higher socioeconomic level may make it less representative of other African countries in terms of sources and characteristics of air pollution mixture, individual exposure, and the demographics more susceptible to the effects of air pollution on health. Additionally, 2 studies focused on private hospitals in Cape Town,^27^^,^^29^ which introduces an additional bias in the representation of the African population, given that only a small percentage of South Africans (13%) have access to private health care/health insurance, and the demographics and lifestyles of these individuals will vary considerably from that of other regions of Africa.^55^

While these findings offer valuable insights into urban contexts, they also present a significant research gap regarding the impact of air pollution on cardiovascular health in other areas of Africa, especially rural areas. It is essential to recognize that air pollution is not solely confined to urban areas; different locations may have varying pollutants with distinct health effects, such as O_3_ or pollution derived from crop burning.^56^ Considering that a substantial proportion of the population of Africa resides in scattered remote rural areas, these individuals may also be particularly vulnerable in terms of health and access to health care. Expanding our research focus beyond urban centers and into rural areas is essential for developing a more holistic understanding of air pollution's health impacts across diverse African landscapes. This broader perspective will enable the formulation of more effective policies and interventions aimed at mitigating the adverse effects of air pollution and promoting public health equity throughout the continent. Furthermore, it is worth noting that indoor air pollution, often stemming from solid fuel use, is significant in Africa. While beyond the scope of this review, it remains a critical consideration, particularly in rural settings where biomass burning, both domestic and from agricultural practices, might contribute to poor air quality.

In addition, desert dust is a notable contributor to air pollution, yet its impact in the African setting is relatively understudied. Although our review did not cover this aspect, it is important to acknowledge that the health effects of desert dust remain a point of scientific discussion. While this source of PM is often larger in size than combustion-derived PM and may have a lesser degree of toxicity in laboratory conditions, a growing literature suggests that it still exerts detrimental effects on health, especially through interaction and adsorption of anthropogenic chemicals on its surface.^57^ Given that Saharan dust can generate remarkably elevated levels of PM over long distances spanning several countries, there is an unmet need for further research on the contribution of desert dust on health outcomes in Africa, and the implications of policies and interventions designed around mass metric of PM rather than defined PM sources.

### Implications for public health and policy

The implications of the reviewed literature extend beyond academia, carrying substantial weight for public health policies and interventions. As we observe the growing burden of CVDs in African populations, exacerbated by the concurrent rise in air pollution levels,^58^ there is an urgent need for evidence-based policies and interventions that are tailored to be effective for African communities. This scoping review serves as a foundational step in identifying the intricate relationship between air quality and cardiovascular health in Africa and could provide a basis for bolstering discussions on policies proposed in this region. The identified gaps and disparities underscore the importance of prioritizing air quality monitoring, implementing targeted public health campaigns, and adopting sustainable practices that reduce pollutant emissions. Our findings describe the current state of knowledge and research gaps in this field and should inform future epidemiological studies and policy interventions to mitigate air pollution and protect cardiovascular health in Africa.

### Study limitations

This scoping review has several limitations. First, the search was limited to PubMed, a comprehensive database for health studies. While PubMed likely captures most relevant articles in peer-reviewed journals, studies indexed in other databases might have been missed. Second, the search strategy focused solely on original articles published in peer-reviewed journals, potentially excluding unpublished studies or reports in non-indexed journals. Third, the number of studies included was relatively small, which may limit the generalizability of our findings. This reflects the overall paucity of research on air pollution and cardiovascular health in Africa. Fourth, due to our focus on CVDs, we did not explore the impact of air pollution on other health conditions in Africa, or subclinical endpoints and risk factors such as hypertension. Fifth, while this review primarily focused on CVD as an umbrella term, as used in most included studies, it is acknowledged that the CVD spectrum in African populations differs significantly from other regions. The use of the umbrella term “CVD” was intended to maximize the identification of studies containing relevant information. However, as additional studies are performed, it may be necessary to consider the most prevalent CVD conditions in Africa to better establish the risk of air pollution in different demographics in different regions of Africa, and tailor health advice accordingly. Finally, most studies included in this review were conducted in urban settings, which may not fully capture the exposure profiles and health impacts experienced in rural areas. While less populous than urban locations, rural regions will account for a large proportion of the Africa population overall and have different characteristics of air pollution exposure. Further studies are warranted to comprehensively assess the full health burden attributable to air pollution across different environments and the continent at large.

### Research recommendations

There is an urgent need to prioritize Africa as a focal point for research to identify the health effects of air pollution in this often-overlooked continent. Research topics should include the following. 1) Information of air pollution characteristics: a better monitoring the criteria air pollutants in different regions of Africa; a more detailed characterization of the sources of air pollution; spatiotemporal changes of different air pollutants within the air pollution mixture; further characterization of the physiochemical characteristics of PM_2.5_ in different African regions. 2) Establishing exposure to air pollutants in different African settings, including urban and rural settings; domestic exposure to indoor air pollution beyond metrics of solid fuel use; a greater understanding of different exposures across communities, such as gender differences in exposure and exposure risk in susceptible individuals. 3) Further epidemiological studies, of various scales, measuring health outcomes; including both “hard” clinical outcomes such as hospital admissions and mortality, but also out-of-hospital events and subclinical outcomes, given that a significant proportion of the effects of air pollution are likely to be missed by hospital and health care records if individuals are unable or do not seek formal medical treatment. Additionally, it is crucial to focus on the unique CVD spectrum in Africa, including the disproportionate burden of hypertensive heart disease, stroke, ischemic heart disease, rheumatic heart disease, and cardiomyopathies. These conditions may have different etiologies and responses to air pollution compared to other regions.^9^ 4) Understanding both short- and long-term interactions between air pollution and cardiovascular health is required, and to establish whether the dose-response relationship for health outcomes are similar to other regions of the world. 5) Establishing the efficacy of different mitigation strategies to reduce emissions and exposure, and tailored advice on air pollution avoidance for susceptible individuals. Finally, in the absence of sufficient ground monitoring data for air pollutants, there is a role for low-cost air pollution monitors as a cost-effective and scalable means for expanding monitoring and exposure in a way that could be linked to cardiovascular parameters in human studies in Africa.

## Conclusions

We found a remarkable paucity of studies looking at air pollution and CVD in Africa, the vast majority confined to the more economically developed South Africa. Nonetheless, within the studies obtained, associations were found between several air pollutants and CVD. This scoping review serves as a foundational step toward a broader and more inclusive research agenda needed to address the significant gaps in epidemiological and burden data in this region. This understanding is vital for informing evidence-based policies and interventions aimed at mitigating the adverse cardiovascular effects of air pollution continent-wide.

Perspectives **COMPETENCY IN PATIENT CARE:** While research is limited, studies show a significant impact of air pollution on cardiovascular health in Africa. Given the potential for high exposures to air pollution in African settings, clinicians should consider air pollution as a contributing risk factor for patients with cardiovascular conditions. Simple behavioral changes to avoid air pollution exposure may reduce the risk of cardiovascular events in susceptible patients.

**TRANSLATIONAL OUTLOOK 1:** Given the potential for diverse and widespread exposure to air pollutants across various African countries, there is an urgent need for improved monitoring of air pollution in Africa. As well as facilitating research, improved monitoring can improve awareness of environmental determinants of health and guide the implementation of air quality measures into public health strategies.

**TRANSLATIONAL OUTLOOK 2:** There are significant research gaps regarding the long-term impact of air pollution on the CVD trajectory in African populations. Future research should focus on the prevention, early detection, and management of CVD attributed to air pollution. Addressing these gaps will enhance our understanding and ability to combat the rising burden of CVD in Africa.

## Funding support and author disclosures

This research was made possible by a grant from the Clean Air Fund awarded to the World Heart Federation. The authors have reported that they have no relationships relevant to the contents of this paper to disclose.
