# Supplementary material for: The Impact of Air Pollution on Cardiovascular Health Outcomes in African Populations: A Scoping Review
Source: JACC Adv. 2024 Nov 13;3(12):101371. doi: 10.1016/j.jacadv.2024.101371 (PMC11733974; doi:10.1016/j.jacadv.2024.101371)
Supplement: Supplemental material [file mmc1.docx]

# Supplemental Appendix

**SUPPLEMENTARY MATERIAL**

CVD comprehensive as MeSH

1. "Cardiovascular Diseases"[Mesh]

CVD restricted as MeSH

1 restricted. "Cardiovascular Diseases"[Mesh:noexp] OR "Heart Failure"[Mesh] OR "Stroke"[Mesh] OR "Myocardial Infarction"[Mesh]:

CVD as Title/Abstract

2. ((("Cardiovascular Diseas*"[Title/Abstract]) OR ("Stroke"[Title/Abstract])) OR ("Myocardial Infarction"[Title/Abstract])) OR ("Heart Failure"[Title/Abstract]):

Air Pollution comprehensive as MeSH

3. "Air Pollution"[Mesh] OR "Air Pollutants"[Mesh] OR "Particulate Matter"[Mesh] OR "Nitrogen Dioxide"[Mesh] OR "Ozone"[Mesh] OR "Sulfur Dioxide"[Mesh] OR "Ammonia"[Mesh]:

Air Pollution restricted as MeSH

3 restricted.  "Air Pollution"[Mesh:noexp] OR “[Air Pollution, Indoor](https://www.ncbi.nlm.nih.gov/mesh/68016902)” [Mesh] OR "Air Pollutants” [Mesh:noexp] OR "Particulate Matter"[Mesh] OR “[Coal Ash](https://www.ncbi.nlm.nih.gov/mesh/68060729)” [Mesh] OR “[Dust](https://www.ncbi.nlm.nih.gov/mesh/68004391)” [Mesh] OR “[Cosmic Dust](https://www.ncbi.nlm.nih.gov/mesh/68019443)” [Mesh] OR “[Smog](https://www.ncbi.nlm.nih.gov/mesh/68012905)” [Mesh] OR “[Smoke](https://www.ncbi.nlm.nih.gov/mesh/68012906)” [Mesh] OR “[Soot](https://www.ncbi.nlm.nih.gov/mesh/68053260)” [Mesh] OR "Nitrogen Dioxide"[Mesh] OR "Ozone"[Mesh] OR "Sulfur Dioxide"[Mesh] OR "Ammonia"[Mesh]:

Air Pollution as Title/Abstract

4. "Air Pollution"[Title/Abstract] OR "Air Pollutant*"[Title/Abstract] OR "Particulate Matter"[Title/Abstract] OR "Nitrogen Dioxide"[Title/Abstract] OR "Ozone"[Title/Abstract] OR "Sulfur Dioxide"[Title/Abstract] OR "Sulphur Dioxide"[Title/Abstract] OR "Ammonia"[Title/Abstract] OR " PM"[Title/Abstract] OR "PM10"[Title/Abstract] OR "PM2.5"[Title/Abstract] OR "PM2.5"[Title/Abstract] OR " Ultrafine particle*"[Title/Abstract] OR " Nitrogen dioxide"[Title/Abstract] OR "NO2"[Title/Abstract] OR "nitrogen oxide*"[Title/Abstract] OR "Ozone"[Title/Abstract] OR "Carbon monoxide"[Title/Abstract] OR "sulfur dioxide"[Title/Abstract] OR "Black carbon"[Title/Abstract] OR "Organic carbon"[Title/Abstract] OR "Ammonia"[Title/Abstract] OR " Ammonium"[Title/Abstract] OR " Organic dust "[Title/Abstract] OR "aerosol"[Title/Abstract] OR "inorganic dust"[Title/Abstract] OR " Volatile organic carbon "[Title/Abstract] OR "Semi-volatile organic carbon"[Title/Abstract] OR "traffic"[Title/Abstract]:

5. 1 OR 2:

5bis. 1 restricted OR 2:

6. 3 OR 4:

6bis. 3 restricted OR 4:

7. 5 AND 6:

7bis.  5bis AND 6bis:

Africa  as MeSH and Title/Abstract

8. "Africa"[MeSH Terms] OR "Africa"[Title/Abstract] OR “Africa South of the Sahara” [Title/Abstract]  OR “Africa, Central” [Title/Abstract] OR “[Africa, Eastern](https://www.ncbi.nlm.nih.gov/mesh/68000351)” [Title/Abstract] OR “[Africa, Southern](https://www.ncbi.nlm.nih.gov/mesh/68000353)” [Title/Abstract]  OR “[Africa, Western](https://www.ncbi.nlm.nih.gov/mesh/68000354)” [Title/Abstract] OR “Africa, Northern” [Title/Abstract]  OR “[Algeria](https://www.ncbi.nlm.nih.gov/mesh/68000462)” [Title/Abstract] OR “[Egypt](https://www.ncbi.nlm.nih.gov/mesh/68004534)” [Title/Abstract] OR “Libya” [Title/Abstract] OR “[Morocco](https://www.ncbi.nlm.nih.gov/mesh/68009018)” [Title/Abstract] OR “[Tunisia](https://www.ncbi.nlm.nih.gov/mesh/68014416)” [Title/Abstract] OR “Africa, Central” [Title/Abstract]  OR “Cameroon” [Title/Abstract] OR “Central African Republic” [Title/Abstract]  OR “Chad” [Title/Abstract]  OR “[Congo](https://www.ncbi.nlm.nih.gov/mesh/68003223)” [Title/Abstract] OR “[Democratic Republic of the Congo](https://www.ncbi.nlm.nih.gov/mesh/68015023)” [Title/Abstract] OR “[Equatorial Guinea](https://www.ncbi.nlm.nih.gov/mesh/68016703)” [Title/Abstract]  OR “[Gabon](https://www.ncbi.nlm.nih.gov/mesh/68005681)” [Title/Abstract] OR “[Sao Tome and Principe](https://www.ncbi.nlm.nih.gov/mesh/2023082)” [Title/Abstract]  OR “[Africa, Eastern](https://www.ncbi.nlm.nih.gov/mesh/68000351)” [Title/Abstract]  OR “[Burundi](https://www.ncbi.nlm.nih.gov/mesh/68002063)” [Title/Abstract] OR “[Comoros](https://www.ncbi.nlm.nih.gov/mesh/68017507)” [Title/Abstract]  OR “[Djibouti](https://www.ncbi.nlm.nih.gov/mesh/68015730)” [Title/Abstract] OR “[Eritrea](https://www.ncbi.nlm.nih.gov/mesh/68019017)” [Title/Abstract]  OR “[Ethiopia](https://www.ncbi.nlm.nih.gov/mesh/68005002)” [Title/Abstract] OR “[Kenya](https://www.ncbi.nlm.nih.gov/mesh/68007630)” [Title/Abstract] OR “[Madagascar](https://www.ncbi.nlm.nih.gov/mesh/68008270)” [Title/Abstract] OR “[Rwanda](https://www.ncbi.nlm.nih.gov/mesh/68012432)” [Title/Abstract] OR “[Seychelles](https://www.ncbi.nlm.nih.gov/mesh/68012750)” [Title/Abstract]  OR “[Somalia](https://www.ncbi.nlm.nih.gov/mesh/68012998)” [Title/Abstract]  OR “[South Sudan](https://www.ncbi.nlm.nih.gov/mesh/2009786)” [Title/Abstract] OR “[Sudan](https://www.ncbi.nlm.nih.gov/mesh/68013397)” [Title/Abstract] OR “[Tanzania](https://www.ncbi.nlm.nih.gov/mesh/68013636)” [Title/Abstract] OR “[Uganda](https://www.ncbi.nlm.nih.gov/mesh/68014454)” [Title/Abstract]  OR “[Africa, Southern](https://www.ncbi.nlm.nih.gov/mesh/68000353)” [Title/Abstract] OR “[Angola](https://www.ncbi.nlm.nih.gov/mesh/68000810)” [Title/Abstract] OR “[Botswana](https://www.ncbi.nlm.nih.gov/mesh/68001902)” [Title/Abstract] OR “[Eswatini](https://www.ncbi.nlm.nih.gov/mesh/68013541)” [Title/Abstract] OR “[Lesotho](https://www.ncbi.nlm.nih.gov/mesh/68007927)” [Title/Abstract]  OR “[Malawi](https://www.ncbi.nlm.nih.gov/mesh/68008295)” [Title/Abstract] OR “[Mozambique](https://www.ncbi.nlm.nih.gov/mesh/68009073)” [Title/Abstract]  OR “[Namibia](https://www.ncbi.nlm.nih.gov/mesh/68009276)” [Title/Abstract] OR “[South Africa](https://www.ncbi.nlm.nih.gov/mesh/68013019)” [Title/Abstract]  OR “[Zambia](https://www.ncbi.nlm.nih.gov/mesh/68015024)” [Title/Abstract]  OR “[Zimbabwe](https://www.ncbi.nlm.nih.gov/mesh/68015030)” [Title/Abstract] OR “[Africa, Western](https://www.ncbi.nlm.nih.gov/mesh/68000354)” [Title/Abstract] OR “[Benin](https://www.ncbi.nlm.nih.gov/mesh/68001541)” [Title/Abstract]  OR “[Burkina Faso](https://www.ncbi.nlm.nih.gov/mesh/68002050)” [Title/Abstract] OR “[Cabo Verde](https://www.ncbi.nlm.nih.gov/mesh/68055726)” [Title/Abstract] OR “[Cote d'Ivoire](https://www.ncbi.nlm.nih.gov/mesh/68007560)” [Title/Abstract] OR “[Gambia](https://www.ncbi.nlm.nih.gov/mesh/68005714)” [Title/Abstract] OR “[Ghana](https://www.ncbi.nlm.nih.gov/mesh/68005869)” [Title/Abstract] OR “[Guinea](https://www.ncbi.nlm.nih.gov/mesh/68016701)” [Title/Abstract] OR “[Guinea-Bissau](https://www.ncbi.nlm.nih.gov/mesh/68006169)” [Title/Abstract] OR “[Liberia](https://www.ncbi.nlm.nih.gov/mesh/68007988)” [Title/Abstract] OR “[Mali](https://www.ncbi.nlm.nih.gov/mesh/68008302)” [Title/Abstract] OR “[Mauritania](https://www.ncbi.nlm.nih.gov/mesh/68008435)” [Title/Abstract] OR “[Niger](https://www.ncbi.nlm.nih.gov/mesh/68009548)” [Title/Abstract] OR “[Nigeria](https://www.ncbi.nlm.nih.gov/mesh/68009549)” [Title/Abstract]  OR “[Senegal](https://www.ncbi.nlm.nih.gov/mesh/68012675)” [Title/Abstract]  OR “[Sierra Leone](https://www.ncbi.nlm.nih.gov/mesh/68012807)” [Title/Abstract]  OR “[Togo](https://www.ncbi.nlm.nih.gov/mesh/68014037)” [Title/Abstract] OR “[Cameroon](https://www.ncbi.nlm.nih.gov/mesh/68002163)” [Title/Abstract]  OR “[Central African Republic](https://www.ncbi.nlm.nih.gov/mesh/68002488)” [Title/Abstract] OR “[Chad](https://www.ncbi.nlm.nih.gov/mesh/68002596)” [Title/Abstract]  OR “[Congo](https://www.ncbi.nlm.nih.gov/mesh/68003223)” [Title/Abstract] OR “[Democratic Republic of the Congo](https://www.ncbi.nlm.nih.gov/mesh/68015023)” [Title/Abstract] OR “[Equatorial Guinea](https://www.ncbi.nlm.nih.gov/mesh/68016703)” [Title/Abstract]  OR “[Gabon](https://www.ncbi.nlm.nih.gov/mesh/68005681)” [Title/Abstract] OR “[Sao Tome and Principe](https://www.ncbi.nlm.nih.gov/mesh/2023082)” [Title/Abstract] OR “[Burundi](https://www.ncbi.nlm.nih.gov/mesh/68002063)” [Title/Abstract] OR “[Comoros](https://www.ncbi.nlm.nih.gov/mesh/68017507)” [Title/Abstract] OR “[Djibouti](https://www.ncbi.nlm.nih.gov/mesh/68015730)” [Title/Abstract] OR “[Eritrea](https://www.ncbi.nlm.nih.gov/mesh/68019017)” OR “[Ethiopia](https://www.ncbi.nlm.nih.gov/mesh/68005002)” OR “[Kenya](https://www.ncbi.nlm.nih.gov/mesh/68007630)” OR “[Madagascar](https://www.ncbi.nlm.nih.gov/mesh/68008270)” OR “[Rwanda](https://www.ncbi.nlm.nih.gov/mesh/68012432)” OR “[Seychelles](https://www.ncbi.nlm.nih.gov/mesh/68012750)” [Title/Abstract]  OR “[Somalia](https://www.ncbi.nlm.nih.gov/mesh/68012998)” [Title/Abstract] OR “[South Sudan](https://www.ncbi.nlm.nih.gov/mesh/2009786)” [Title/Abstract] OR “[Sudan](https://www.ncbi.nlm.nih.gov/mesh/68013397)” [Title/Abstract]  OR “[Tanzania](https://www.ncbi.nlm.nih.gov/mesh/68013636)” [Title/Abstract] OR “[Uganda](https://www.ncbi.nlm.nih.gov/mesh/68014454)” [Title/Abstract] OR “[Angola](https://www.ncbi.nlm.nih.gov/mesh/68000810)” [Title/Abstract] OR “[Botswana](https://www.ncbi.nlm.nih.gov/mesh/68001902)” [Title/Abstract] OR “[Eswatini](https://www.ncbi.nlm.nih.gov/mesh/68013541)” [Title/Abstract] OR “[Lesotho](https://www.ncbi.nlm.nih.gov/mesh/68007927)” [Title/Abstract] OR “[Malawi](https://www.ncbi.nlm.nih.gov/mesh/68008295)” [Title/Abstract] OR “[Mozambique](https://www.ncbi.nlm.nih.gov/mesh/68009073)” [Title/Abstract] OR “[Namibia](https://www.ncbi.nlm.nih.gov/mesh/68009276)” [Title/Abstract]  OR “[South Africa](https://www.ncbi.nlm.nih.gov/mesh/68013019)” [Title/Abstract] OR “[Zambia](https://www.ncbi.nlm.nih.gov/mesh/68015024)” [Title/Abstract] OR “[Zimbabwe](https://www.ncbi.nlm.nih.gov/mesh/68015030)” [Title/Abstract] OR “[Benin](https://www.ncbi.nlm.nih.gov/mesh/68001541)” [Title/Abstract] OR “[Burkina Faso](https://www.ncbi.nlm.nih.gov/mesh/68002050)” [Title/Abstract] OR “[Cabo Verde](https://www.ncbi.nlm.nih.gov/mesh/68055726)” [Title/Abstract] OR “[Cote d'Ivoire](https://www.ncbi.nlm.nih.gov/mesh/68007560)” [Title/Abstract]  OR “[Gambia](https://www.ncbi.nlm.nih.gov/mesh/68005714)” [Title/Abstract] OR “[Ghana](https://www.ncbi.nlm.nih.gov/mesh/68005869)” [Title/Abstract] OR “[Guinea](https://www.ncbi.nlm.nih.gov/mesh/68016701)” [Title/Abstract]  OR “[Guinea-Bissau](https://www.ncbi.nlm.nih.gov/mesh/68006169)” [Title/Abstract]  OR “[Liberia](https://www.ncbi.nlm.nih.gov/mesh/68007988)” [Title/Abstract] OR “[Mali](https://www.ncbi.nlm.nih.gov/mesh/68008302)” [Title/Abstract] OR “[Mauritania](https://www.ncbi.nlm.nih.gov/mesh/68008435)” [Title/Abstract] OR “[Niger](https://www.ncbi.nlm.nih.gov/mesh/68009548)” [Title/Abstract] OR “[Nigeria](https://www.ncbi.nlm.nih.gov/mesh/68009549)” [Title/Abstract] OR “[Senegal](https://www.ncbi.nlm.nih.gov/mesh/68012675)” [Title/Abstract] OR “[Sierra Leone](https://www.ncbi.nlm.nih.gov/mesh/68012807)” [Title/Abstract] OR “[Togo](https://www.ncbi.nlm.nih.gov/mesh/68014037)” [Title/Abstract] OR “[Algeria](https://www.ncbi.nlm.nih.gov/mesh/68000462)” [Title/Abstract] OR “[Egypt](https://www.ncbi.nlm.nih.gov/mesh/68004534)” [Title/Abstract] OR “[Libya](https://www.ncbi.nlm.nih.gov/mesh/68008002)” [Title/Abstract]  OR “[Morocco](https://www.ncbi.nlm.nih.gov/mesh/68009018)” [Title/Abstract] OR “[Tunisia](https://www.ncbi.nlm.nih.gov/mesh/68014416)” [Title/Abstract] OR “[Algeria](https://www.ncbi.nlm.nih.gov/mesh/68000462)” [Title/Abstract] OR “[Egypt](https://www.ncbi.nlm.nih.gov/mesh/68004534)” [Title/Abstract] OR “[Libya](https://www.ncbi.nlm.nih.gov/mesh/68008002)” [Title/Abstract] OR “[Morocco](https://www.ncbi.nlm.nih.gov/mesh/68009018)” [Title/Abstract] OR “[Tunisia](https://www.ncbi.nlm.nih.gov/mesh/68014416)” [Title/Abstract]: 601,448

9. 7 AND 8

9bis. 7bis AND 8

**Quality assessment table**

| Author | Pollutant measurement quality score (0-1 point) | CVD Validity | Adjustment quality score (0-3 points) | Total |
| --- | --- | --- | --- | --- |
| Wichmann 2012 | 1 | 1 | 3 | 5 |
| Lin 2017 | n/a | n/a | n/a | n/a |
| Roomaney 2022 | 0 | 0 | 0 | 0 |
| Adebayo 2022 | 1 | 1 | 3 | 5 |
| Lokotola 2020 | 1 | 1 | 3 | 5 |
| Adebayo 2022 | 1 | 1 | 3 | 5 |

**RISK assessment for cross sectional study**

| 1. Was the research question or objective in this paper clearly stated? - Y, fair |  |  |  |
| --- | --- | --- | --- |
| 2. Was the study population clearly specified and defined? - Y, good |  |  |  |
| 3. Was the participation rate of eligible persons at least 50%? - n/a |  |  |  |
| 4. Were all the subjects selected or recruited from the same or similar populations (including the same time period)? Were inclusion and exclusion criteria for being in the study prespecified and applied uniformly to all participants? - yes, |  |  |  |
| 5. Was a sample size justification, power description, or variance and effect estimates provided? - n/a |  |  |  |
| 6. For the analyses in this paper, were the exposure(s) of interest measured prior to the outcome(s) being measured? - Y- good |  |  |  |
| 7. Was the timeframe sufficient so that one could reasonably expect to see an association between exposure and outcome if it existed? - yes, good |  |  |  |
| 8. For exposures that can vary in amount or level, did the study examine different levels of the exposure as related to the outcome (e.g., categories of exposure, or exposure measured as continuous variable)? - na |  |  |  |
| 9. Were the exposure measures (independent variables) clearly defined, valid, reliable, and implemented consistently across all study participants? - y, fair to poor |  |  |  |
| 10. Was the exposure(s) assessed more than once over time? -no, n/a |  |  |  |
| 11. Were the outcome measures (dependent variables) clearly defined, valid, reliable, and implemented consistently across all study participants? - yes, poor to fair |  |  |  |
| 12. Were the outcome assessors blinded to the exposure status of participants? - n/a |  |  |  |
| 13. Was loss to follow-up after baseline 20% or less? - n/a |  |  |  |
| 14. Were key potential confounding variables measured and adjusted statistically for their impact on the relationship between exposure(s) and outcome(s)? - yes, good |  |  |  |

**ICD-10 codes**

The following ICD codes were used for cardiovascular diseases (CVDs) in the study:

I01-I01.9 Acute rheumatic fever

I05-I09 Chronic rheumatic heart diseases

I11- I13.9 Hypertensive diseases excluding primary hypertension and secondary hypertension

I20-I25 Ischemic heart diseases

I26-I28 Pulmonary heart diseases

I30-I52 Other forms of heart diseases

I60-I69 Cerebrovascular diseases

I70-I79 Diseases of arteries, arterioles and capillaries
